# Asymmetric Dimethylarginine (ADMA) and Symmetric Dimethylarginine (SDMA) Concentrations in Patients with Obesity and the Risk of Obstructive Sleep Apnea (OSA)

**DOI:** 10.3390/jcm8060897

**Published:** 2019-06-23

**Authors:** Yana Arlouskaya, Ada Sawicka, Marek Głowala, Joanna Giebułtowicz, Natalia Korytowska, Marek Tałałaj, Grażyna Nowicka, Małgorzata Wrzosek

**Affiliations:** 1 Department of Biochemistry and Pharmacogenomics, Faculty of Pharmacy and Laboratory of Biochemistry and Clinical Chemistry at the Preclinical Research Center, Medical University of Warsaw, 02-097 Warsaw, Poland; jana.orlowskaya@gmail.com (Y.A.); mglowala@wum.edu.pl (M.G.); grazyna.nowicka@wum.edu.pl (G.N.); 2 Department of Family Medicine, Internal Medicine and Metabolic Bone Diseases, Medical Centre of Postgraduate Education, 00-416 Warsaw, Poland; asaw73@o2.pl (A.S.); marektalalaj@op.pl (M.T.); 3 Department of Bioanalysis and Drug Analysis, Medical University of Warsaw, 02-097 Warsaw, Poland; jgiebultowicz@wum.edu.pl (J.G.); nkorytowska@wum.edu.pl (N.K.)

**Keywords:** obesity, predictors of obstructive sleep apnea, dimethylarginines

## Abstract

Asymmetric dimethylarginine (ADMA) and symmetric dimethylarginine (SDMA) are endogenous inhibitors of nitric oxide (NO) synthesis, and play a critical role in the process of endothelial dysfunction, and are considered markers of oxidative stress. The aim of the present study was to explore relationships between ADMA and/or SDMA and the occurrence of OSA in obese patients as well as the effect of the endothelial nitric oxide synthase (eNOS) gene polymorphism, which may modify the influence of ADMA or SDMA on NO production. A total of 518 unrelated obese subjects were included in this study. Body weight, height and blood pressure were measured and data on self-reported smoking status were collected. Obstructive sleep apnea (OSA) was assessed by the apnea hypopnea index (AHI). Blood samples were collected to measure serum concentrations of glucose, total cholesterol, LDL-cholesterol, HDL-cholesterol, triglycerides, creatinine, HbA1c (%), folic acid, vitamin B_12_, C-reactive protein (CRP), aspartate aminotransferase (ASP), alanine aminotransferase (ALT) and IL-6 by routine methods. The *NOS3* gene G894T and 4a/4b polymorphisms were detected by polymerase chain reaction-restriction fragment length polymorphism (PCR-RFLP) analysis. ADMA, SDMA and arginine concentrations were assessed simultaneously using liquid chromatography coupled with tandem mass spectrometry (LC-MS/MS) method. Adjusted multivariate logistic regression analysis showed a significant association between the occurrence of OSA and high serum ADMA levels, BMI above 40, age > 43 years, hypertension and male sex. Heterozygotes for the G894T eNOS polymorphism have the lowest serum concentrations of ADMA and SDMA, while no effect of the 4a/4b variants was observed. The results indicate that OSA in obese individuals can coexist with high ADMA levels, which appear as a potential OSA predictor.

## 1. Introduction

Obstructive sleep apnea (OSA) is a chronic, sleep-related breathing disorder with increasing prevalence. Obesity is one of the key risk factors for OSA, and untreated OSA increases the risk of arterial hypertension, coronary artery disease, arrhythmia, heart failure and cerebrovascular diseases commonly associated with obesity. It can be assumed that OSA incidence will continue to rise along with the ongoing obesity epidemic. On the other hand, available data indicate that, currently, most people with OSA are undiagnosed and untreated [1]. Therefore, biomarkers that could help to identify obese patients at higher risk of OSA could improve early OSA diagnosis.

**Scheme 1:**
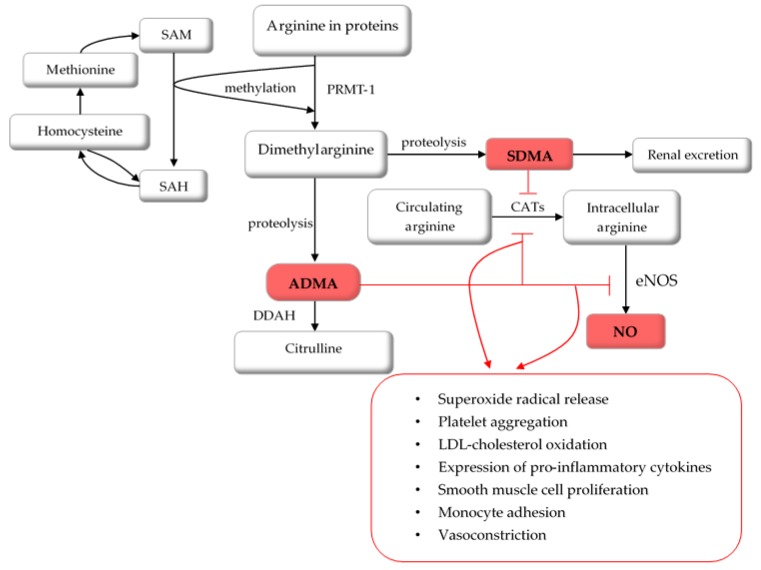
The effect of ADMA and SDMA on NO production. ADMA, asymmetric dimethylarginine; SDMA, symmetric dimethylarginine; NO, nitric oxide; eNOS, endothelial nitric oxide synthase; DDAH, dimethylarginine dimethylaminohydrolase; PRMT, protein arginine N-methyltransferase; SAM, S-adenosylmethionine; SAH, S-adenosylhomocysteine; CATs, cationic amino acid transporter.

Asymmetric dimethylarginine (ADMA) is a methylated derivative of the amino acid L-arginine, which is receiving increased attention as a cardiovascular risk factor. As a structural analog of L-arginine, ADMA can directly inhibit nitric oxide (NO) synthesis by competitive binding to NO synthases, resulting in decreased NO production in blood vessels and other tissues. ADMA also enhances nitric oxide synthase (NOS) uncoupling and the production of reactive oxidative species (ROS), such as superoxide anion (O^2−^) and peroxynitrite (ONOO^−^), which could further reduce the cardiovascular NO bioavailability [2]. Existing experimental and clinical evidence indicates that even a small change in ADMA levels significantly affects the intensity of nitric oxide production [3]. Symmetric dimethylarginine (SDMA) is another methylated analogue of L-arginine found in humans. ADMA and SDMA are generated from proteins, which are methylated on arginine residues by protein arginine N-methyltransferases (PRMTs). PRMTs utilize SAM (S-adenosylmethionine) as a methyl donor and generate SAH (S-adenosylhomocysteine) and, ultimately, homocysteine as a byproduct [4]. ADMA is either eliminated by renal excretion or degraded by dimethylarginine dimethylaminohydrolase (DDAH). In contrast to its stereoisomer, SDMA is almost completely eliminated with urine [5]. Both SDMA and ADMA may reduce NO synthesis indirectly by inhibiting the cellular uptake of L-arginine, the NO precursor (Scheme 1). Nitric oxide is involved in many biological processes. It causes the relaxation of smooth muscle cells and the subsequent vasodilatation of blood vessels; prevents smooth muscle cells proliferation and migration; reduces platelet adhesion and aggregation; and reduces monocyte stickiness. It also exerts anti-inflammatory activity and reduces oxidation of LDL-cholesterol [2]. In endothelium, NO is synthesized by specific NO synthase (eNOS). Polymorphisms of the eNOS gene are among the factors affecting nitric oxide synthase activity and basal NO production [5]. Disturbances in NO production and bioavailability have been recognized to increase the risk of developing hypertension and other cardiovascular diseases commonly associated with OSA. Clinical and experimental evidence indicate that ADMA and SDMA are involved in the endothelial dysfunction, oxidative stress, and inflammation [6,7] and are associated with an enhanced risk of cardiovascular diseases [2,4,8]. However, there is a lack of data describing potential associations between serum ADMA and SDMA concentrations and BMI or OSA. There is also no data on the interaction between ADMA, SDMA, arginine and the nitric oxide synthase gene polymorphism. Thus, the aim of the present study was to explore relationships between the occurrence of OSA in individuals with obesity and ADMA and/or SDMA levels, as well as the effect of the endothelial nitric oxide synthase (eNOS) gene polymorphism, which may modify the influence of ADMA or SDMA on NO production.

## 2. Material and Methods

### 2.1. Study Population

A total of 518 unrelated individuals with obesity were enrolled in this study. All subjects were consecutively recruited on the basis of clinical investigation between September 2013 and September 2018 from patients who had been admitted to the Orlowski Hospital in Warsaw. 

The study was carried out in accordance with the principles of the Declaration of Helsinki. The whole study protocol and the consent procedure were approved by the Institutional Bioethics Committees (KB/127/2012 and KB/67/2017 at the Medical University of Warsaw; and 7/PB/2015 at the Medical Centre of Postgraduate Education). Written informed consent was obtained from each participant after a full explanation of the study.

A detailed clinical history, and a full physical examination, was obtained for each patient. All participants completed a questionnaire concerning smoking habits. Smoking status was categorized as never a smoker, current smoker, and former smoker. In all subjects, anthropometric measurements (body weight, and height) were taken and body mass index (BMI) was calculated as the ratio of weight (kilograms) to the square of height (meters). The data on DXA-derived measures of total body fat (fat mass expressed as % fat mass and kg) was available for the whole group of participants. Obesity was classified according to World Health Organization criteria, and subjects with BMI ≥30 kg/m^2^ were considered obese. Participants were classified as being hypertensive if they had an average blood pressure ≥140/90 mm Hg, assessed as previously described [9], or by a previous diagnosis of hypertension and they were on hypertensive medication at the time of the interview. Patients were classified as diabetics based on the review of medical records (previous diagnosis of diabetes by a physician, and current use of glucose-lowering medications) and confirmed by current medical examination. The diagnosis was made using criteria consistent with those proposed by the American Diabetes Association [10] (an average fasting glucose concentration ≥126 mg/dL on two occasions, and/or 2 h glucose >200 mg/dL during an oral-glucose-tolerance test, and/or a casual glucose >200 mg/dL). Participants were classified as having dyslipidemia if they had received a diagnosis from a physician according to the National Cholesterol Education Program-Adult Treatment Panel III (ATP III) guidelines [11] and/or reported the use of lipid lowering medications. All participants underwent standard overnight assessment (polysomnography, PSG) to evaluate the presence of obstructive sleep apnea (OSA). OSA was assessed by the apnea hypopnea index (AHI), which is the number of complete (apneas) or incomplete (hypopneas) obstructive events per hour of sleep, and defined as AHI ≥5 [12].

### 2.2. Measurements of Biomarkers

Overnight peripheral fasting blood samples were taken from all subjects, and the serum was either isolated and used for analyses, or stored at −80 °C. All samples were analyzed by specialized clinical laboratory medical personnel. The laboratory analyses included measurements of total cholesterol, high-density lipoprotein cholesterol (HDL-C), low-density lipoprotein-cholesterol (LDL-C), triglycerides, glucose, creatinine (CRE), HbA1c(%), folic acid, vitamin B_12_, C-reactive protein (CRP), aspartate aminotransferase (ASP), alanine aminotransferase (ALT) and erythrocyte sedimentation rate (ESR). Serum levels of interleukine 6 (IL-6) were determined by enzyme-linked immunosorbent assay (ELISA), using the Diaclone Human IL-6 High Sensitivity ELISA kit (Diaclone SAS, Besancon Cedex, France). The estimated glomerular filtration rate (eGFR) was calculated by the modification of diet in renal disease (MDRD) and the chronic kidney disease epidemiology collaboration (CKD-EPI) equations [13]:

MDRD_186_: eGFR (mL/min/1.73 m^2^) = 186 × (serum creatinine, mg/dL)^−1.154^ × (age)^−0.203^ × 0.742 [if female], (1)

CKD EPI: eGFR (mL/min/1.73 m^2^) = 141 × min(serum creatinine, mg/dL/κ, 1)^α^ × max(serum creatinine, mg/dL /κ, 1)^−1.209^ × 0.993^Age^ × 1.018 [if female], (2)

### 2.3. Genotyping of the NOS3 4a/4b and NOS3 G894T Polymorphisms

Genomic DNA was extracted and purified from peripheral blood leukocytes using the Blood Mini genomic DNA kit (A&A Biotechnology, Gdynia, Poland) according to the manufacturer’s instructions and quantified by a UV-Vis spectrophotometer (Quawell Q3000, Quawell Technology Inc., San Jose, CA, USA). The 27-bp repeat polymorphism in intron 4 of the *NOS3* gene (*NOS3* 4a/4b polymorphism) was analyzed by PCR amplification. The amplified products were analyzed by electrophoresis on agarose gel containing ethidium bromide and visualized under UV light (ChemiDoc MP, Bio-Rad, Hercules, CA, USA). The eNOS G894T polymorphism (rs1799983) was evaluated by polymerase chain reaction - restriction fragment length polymorphism (PCR-RFLP) analysis. The detailed information of those sites was the same as previously described [9].

### 2.4. ADMA, SDMA, and Arginine Measurements

ADMA, SDMA and arginine concentrations were assessed simultaneously using liquid chromatography coupled with tandem mass spectrometry (LC-MS/MS).

Instrumental analysis was carried out by liquid chromatography (LC) coupled with a hybrid triple quadrupole/linear ion trap mass spectrometer (QTRAP 4000; AB SCIEX, Framingham, MA, USA). LC analysis was carried out with an Agilent 1260 Infinity (Agilent Technologies, Santa Clara, CA, USA), equipped with a degasser, thermostated autosampler and binary pump, and connected to a mass spectrometer equipped with a turbo ion spray source operated in positive mode. The samples were prepared as follows: 50 μL of plasma was transferred to an Eppendorf tube (1.5 mL), and 200 μL of acetonitrile with the internal standards (Arg-^13^C6, ADMA-D6, final concentration of 7.5 µg mL^−1^ and 155 ng mL^−1^, respectively) was then added. This mixture was mixed on a vortex for 5 min, kept at −20 °C for 20 min and centrifuged at 9300 rcf in 4 °C for 10 min. The supernatant was transferred to the appropriate high-performance liquid chromatography vials. This study used a SeQuant ZIC-HILIC (zwitterionic hydrophilic interaction liquid chromatography) column (50 mm × 2.1 mm, particle size 5 µm) supplied by Merck (Darmstadt, DE). The column was maintained at 40 °C and at a flow rate of 0.5 mL min^−1^. The mobile phases consisted of water solution of 20 mM ammonium acetate as eluent A and acetonitrile with 0.2% formic acid as eluent B. The gradient (%B) was as follows: 0 min 90%; 1 min 90%; 7 min 50%; 8 min 50%. The target compounds were analyzed in multiple reaction monitoring mode. The transitions used for quantitation were *m/z* 203 > 46 and *m/z* 203 > 172 and *m/z* 209 > 77 for ADMA, SDMA and ADMA-D6 and *m/z* 175 > 116 and *m/z* 181 > 121 for Arg and Arg-^13^C6, respectively. The compound parameters, viz. declustering potential (DP), collision energy (CE), entrance potential (EP) and collision cell exit potential (CXP) were: 61 V, 41 V, 10 V, 0 V for ADMA; 61 V, 19 V, 10 V, 10 V for SDMA; 66 V, 45 V, 10 V, 4 V for ADMA-D6; 61 V, 21 V, 10 V, 8 V for Arg and 31 V, 21 V, 10 V, 10 V for Arg-^13^C6. The curtain gas, ion source gas 1, ion source gas 2 and collision gas (all high purity nitrogen) were set at 241 kPa, 207 kPa, 345 kPa and high instrument units, respectively. The ion spray voltage and source temperature were set at 5500 V and 600 °C, respectively.

### 2.5. Statistical Analysis

Continuous variables are presented as the mean values (with the standard deviation) and as median values (with the interquartile range) to allow better comparison with other publications. Categorical variables are reported as frequencies and percentages. The normality of continuous variable distribution was tested with the Shapiro-Wilk test. The parameters measured as continuous variables were compared by ANOVA, Student *t*-test or Kruskall-Wallis tests or Mann-Whitney tests as necessary. Categorical variables were compared using the Chi-square test. Linear regression analysis was used to identify factors associated with serum ADMA concentrations. All variables with a significant univariate association with the outcome measure were included in the final multivariate model. A linear association between parameters was also investigated using either the Pearson correlation coefficient or Spearman’s Rank correlation coefficient (Rho), according to the distribution. Multiple logistic regression was used to assess predictors of OSA. The variables that were significantly related with OSA in univariate analyses were then included in the multivariate logistic regression model. Qualitative variables were coded as 0–1 dummy variables. The results from the logistic regression model are presented as odds ratios (OR) with a 95% confidence interval (CI). The ability of ADMA levels to discriminate between patients with and without OSA was assessed by receiver operating characteristic curves and associated area under the curve (AUC). The optimal cutoff point of ADMA score to discriminate between patients with and without OSA was calculated by determining the value that provided the greatest sum of sensitivity and specificity. All statistical analyses were performed using Statistica software (Statistica version 12.0, StatSoft Inc., Tulsa, OK, USA) and a *p* value < 0.05 was considered statistically significant.

## 3. Results

Demographic and biochemical characteristics of the study participants are expressed in Table 1. The study group consisted of 384 women and 134 men with a mean age of 43.8 ± 11.3 years and an average BMI of 42.6 ± 6.7 kg/m^2^. The majority of participants (62.3%) had a BMI above 40 kg/m^2^, 28.6% had a BMI in the range of 35–39.9, and 9.1% of subjects were categorized as having class I obesity (BMI: 30.0–34.9 kg/m^2^). Altogether, 242 obese subjects with OSA and 276 obese subjects without OSA participated in the study (Table 2). 

Levels of ADMA and SDMA were statistically significantly associated with a number of the biomarkers tested (Table 1), with moderate correlations found only between SDMA and creatinine, and eGFR, with the remaining having weak correlations (i.e., BMI, apnea hypopnea index, folic acid, B_12,_ fat%, CRP, IL-6, age, HbA1c%, glucose and lipids). In addition, both ADMA and SDMA levels were increased in patients with class II and III obesity in comparison to patients with class I obesity (*p* < 0.05, Table 2). In the case of SDMA, however, post-hoc analysis using Tukey’s test did not reach significance.

Patients with hypertension had a higher concentration of serum SDMA and lower concentration of serum arginine when compared to the normotensive group (Table 2), while serum ADMA levels did not differ between patients with and without hypertension. However, an effect of *NOS3* G894T polymorphisms on serum ADMA concentrations was found and the occurrence of the GT genotype was associated with the lowest serum ADMA concentrations. No effect of the 4a/4b *NOS3* variants on ADMA, SDMA and arginine levels was recognized (Table 3).

**Figure 1:**
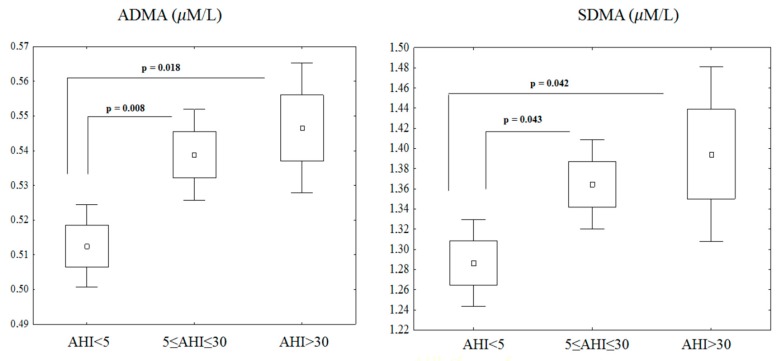
The serum ADMA (µmol/L) and SDMA (µmol/L) concentrations in patients with mild and moderate (5 ≤ AHI ≤ 30) and severe OSA (AHI > 30), and patients without OSA (AHI < 5). The boxplots show the mean, SE (standard error) and 1.96 * SE.

As ADMA and SDMA affect NO synthesis, a relation between serum ADMA and SDMA concentrations and obstructive sleep apnea was also examined in 518 patients who underwent overnight polysomnography (PSG). Based on PSG and according to the apnea-hypopnea index (AHI) we divided patients into 2 groups: OSA (AHI ≥ 5) and non-OSA (AHI < 5). The patients with OSA had significantly higher concentrations of ADMA (0.51 ± 0.09 μmol/L vs. 0.54 ± 0.09 μmol/L) and SDMA (1.29 ± 0.33 μmol/L vs. 1.37 ± 0.32 μmol/L) than patients without OSA (Table 3). The analyses, in which concentrations of ADMA and SDMA in patients with mild and moderate (5 ≤ AHI ≤ 30) and severe OSA (AHI > 30) as well as in patients without OSA (AHI < 5) were compared (Figure 1), suggest that an association between OSA severity and ADMA and SDMA may occur. However, further studies are needed to elucidate this relationship.

Logistic regression analysis revealed that high (>50th percentile) serum ADMA and SDMA concentrations were associated with a higher chance of having OSA (with OR = 1.71; 95% CI: 1.21–2.43 and OR = 1.58; 95% CI: 1.12–2.24, respectively, as presented in Table 4). Individuals with obstructive sleep apnea have recurrent hypoxia at night, which is associated with the collapse of the upper respiratory tract. An important factor leading to the occurrence of obstructive sleep apnea is obesity, as it contributes to the expansion of the structures of the soft tissues in respiratory tract, thereby making a significant contribution to the narrowing of the pharyngeal respiratory tract. In our group, subjects with BMI above 40 had 2.09 times higher odds (OR = 2.09; 95% CI = 1.45–3.02; Table 4) of having OSA than those with BMI in the range of 30–39.9. In the OSA group, 40.5% were male, as opposed to 13% male participants in the non-OSA group (*p* < 0.001, Table 4), and the obese males compared to females had a 4.54-fold higher chance (OR = 4.54; 95% CI = 2.94–7.00) of having OSA (Table 4). The prevalence of hypertension and higher-than-median age was also significantly higher in the OSA group than in the non-OSA group (*p* < 0.001; Table 4). 

The variables significantly associated with OSA in univariable analyses (Model 1), i.e., elevated ADMA and SDMA concentrations, BMI above 40, older age (i.e., higher-than-median age), hypertension, and male sex, were then combined in the multivariable logistic regression model. After adjustment for Model 1, and further for prevalence of diabetes, dyslipidemia, smoking status and eGFR > 60 mL/min/1.73 m^2^ (Model 2), the results for ADMA remained unchanged but the association for SDMA became insignificant. 

Receivers operating characteristic (ROC) curves were also used as a statistical model to evaluate ADMA as a marker of OSA. Upon ROC curve analysis, ADMA levels were found to significantly discriminate between patients with and without OSA, with an AUC of 0.601 (95%CI 0.55–0.65, *p* = 0.0002). The cutoff to predict the presence of AHI ≥ 5 in obese individuals was an ADMA level > 0.46 μmol/L, with a sensitivity of 84% and a specificity of 27%.

## 4. Discussion

Asymmetric dimethylarginine (ADMA), a strong inhibitor of nitric oxide synthase, and symmetric dimethylarginine (SDMA) are toxic amino acids that inhibit nitric oxide (NO) production. The loss of NO production and bioactivity can be associated with development of pathological conditions such as hypertension or obstructive sleep apnea (OSA) [14,15]. Both of these conditions are characterized by an increased expression of pro-inflammatory cytokines and the production of reactive oxygen species (ROS), which are also commonly seen in obesity [16]. Studying the metabolic pathway of these mechanisms may be of relevance to the fact that ADMA is also produced by adipocytes, which express the whole gene set responsible for the biosynthesis and the degradation of ADMA [17]. Accordingly, Sydow et al. [18], who investigated 980 healthy adults, found a positive correlation between ADMA and BMI. This study showed that patients with BMI ≥ 40 had significantly higher concentrations of ADMA than patients with class I obesity. It can be hypothesized that increased ADMA levels and the development of ADMA-related pathologies can, at least in part, be explained by fat mass expansion and impaired synthesis of adipocyte-derived mediators [19].

In this study, serum ADMA and SDMA levels in obese individuals positively correlated with the apnea hypoxia index, and the concentrations of these compounds were higher in patients with OSA than in patients without OSA (AHI < 5). Moreover, the occurrence of high serum ADMA and SDMA levels (>50th percentile) was associated with a higher chance of having OSA. However, in the presence of strong OSA risk factors such as male sex, body mass index, and age, the impact of SDMA was found to be unsubstantial. Nevertheless, our study, for the first time, demonstrated that ADMA levels can discriminate between patients with and without OSA, and ADMA appeared as an independent risk factor for OSA in patients with obesity. Previously ADMA was reported as an independent predictor of angiographic coronary atherosclerosis [20,21,22] and diabetes [23]. 

Obesity is commonly accepted as major factor leading to obstructive sleep apnea. In our study, only subjects with BMI ≥ 30 were included. However, the authors were still able to confirm that patients with BMI above 40 had higher odds of having OSA than those with BMI in the range of 30–39.9. The association between ADMA and OSA was significant after adjustment for potential confounders, such as diabetes, dyslipidemia, smoking status and eGFR > 60 mL/min/1.73 m^2^, the last because the concentrations of ADMA are increased in patients with renal failure [24,25]. Thus, the authors used eGFR to exclude the potential effect of renal failure on the positive association between ADMA and OSA. Moreover, the prevalence of hypertension, male sex and higher-than-median age was significantly higher in the OSA group than in the non-OSA group. These results are in accordance with previous data showing that age, male sex and body mass index were strongly associated with OSA [26,27]. 

Clinical and experimental evidence indicates that ADMA and SDMA are involved in the endothelial dysfunction and inflammation [6,7]. The development of inflammation in OSA was found to be initiated by hypoxia through the activation of the nuclear factor-kappaB (NF-κB) [28]. This transcription factor is responsible for the expression of IL-6. In our study, serum IL-6 concentrations were significantly correlated with serum ADMA and SDMA concentrations. Moreover, our present study showed an association between ADMA and CRP in patients with obesity, confirming previously published data in varying sets of patients [23,29]. Inflammation is commonly associated with increased ROS generation [30]. Under oxidative stress, nitric oxide synthase (NOS) uncoupling occurs and results in increased production of the superoxide anion, which can further react with NO and generate peroxynitrite, a very active free radical. It results in further modulation of the oxidation process [2]. Therefore, elevated concentrations of inflammatory cytokines and ADMA can be linked to oxidative stress and obstructive sleep apnea [31]. The exact mechanism for increased ADMA is not elucidated in detail. In principle, the overall ADMA elevation can be a result of increased ADMA production in response to stresses or increased degradation and turnover of proteins containing methylated arginine, decreased ADMA degradation by DDAH, and reduced ADMA excretion by the kidneys [2].

In our study, inverse relationships were noted between levels of folic acid, ADMA and SDMA, and between vitamin B_12_ and SDMA. Vitamin B_12_ and folic acid are crucial for the production of methyl groups involved in the metabolic pathways of ADMA and SDMA. Folate and vitamin B_12_ deficiency results in accumulation of homocysteine. In some studies, an association between hyperhomocysteinemia and high ADMA or SDMA was identified [32,33,34], but the relationship between increased ADMA concentration and elevated concentration of homocysteine during experimental hyper- and hypohomocysteinemia remains unclear [35,36].

Our study also showed that patients with hypertension had higher concentrations of serum SDMA and lower concentrations of serum arginine in comparison with the normotensive group, while serum ADMA levels were not significantly different. Previously high ADMA levels were reported in individuals with hypertension [37,38]. However, the authors have to emphasize that our group was not a purely hypertensive group as in, for example, the study of Sonmez et al. [38], where young male, non-obese, and newly diagnosed hypertensive individuals were enrolled to prevent any interference of obesity, insulin resistance, hyperglycemia or medication. This study stratified the patient population according to the presence of the endothelial nitric oxide synthase gene G894T and the 4a/4b polymorphisms to assess the potential effects of these polymorphisms on ADMA, SDMA and arginine concentrations. The present study’s results indicated that, among obese patients, an association between the *NOS3* 894 GT genotype and low serum ADMA concentration may occur. With regard to these results, it remains a central piece of the puzzle how the observed effect of eNOS polymorphism on ADMA can be reconciled with changes in ADMA concentrations so modest that they are unlikely to alter NO synthesis significantly. Further studies are needed to confirm the influence of nitric oxide synthase polymorphism on ADMA-related pathologies. 

This study had some limitations. This study has not considered other possible genetic and metabolic factors affecting NO synthesis and bioavailability. Homocysteine concentrations were not measured which could give additional insight into disturbances in ADMA and methyl group metabolism.

In conclusion, serum ADMA concentrations were found to be significantly elevated in patients with OSA and obesity and it was not affected by hypertension, diabetes, smoking status, and renal failure. However, further studies are needed to confirm ADMA as a biomarker for OSA risk in patients with obesity.

## Figures and Tables

**Table 1:** Clinical and biochemical characteristics of the study participants and Spearman Rank correlation analysis with asymmetric dimethylarginine (ADMA), symmetric dimethylarginine (SDMA), arginine, and ratio Arginine/ADMA.

Variables	Mean ± SD	Median (IQR)	* Rho/*p*-Value			
			ADMA	SDMA	Arginine	Ratio Arginine/ADMA
ADMA (µmol/L)	0.5 ± 0.1	0.5 (0.5–0.6)	-	**0.48/<0.0001**	**0.21/<0.0001**	**−0.45/<0.0001**
SDMA (µmol/L)	1.3 ± 0.1	1.3 (1.1–1.5)	**0.48/<0.0001**	-	0.01/0.7894	**−0.31/<0.0001**
Arginine (µmol/L)	105.87 ± 38.9	99.31 (86.11–115.67)	**0.21/<0.0001**	0.01/0.7894	-	**0.73/<0.0001**
Ratio Arg/ADMA	177.3 ± 66.3	165.2 (139.1–197.3)	**−0.45/0.0001**	**−0.31/<0.0001**	**0.73/<0.0001**	-
Age in years	43.8 ± 11.3	43.0 (35.0–53.0)	−0.01/0.8345	**0.19/<0.0001**	**−0.14/0.0016**	**−0.12/0.0076**
Weight (kg)	121.4 ± 23.6	119.0 (103.5–134.0)	**0.17/0.0001**	**0.15/0.0005**	**0.04/0.316**5	−0.07/0.0934
BMI	42.6 ± 6.7	41.6 (37.7–46.4)	**0.23/0.0001**	**0.15/0.0008**	0.04/0.3352	−0.11/0.1070
Fat %	42.8 ± 6.8	44.2 (39.1–47.4)	**0.12/0.0161**	0.07/0.1335	0.03/0.5140	−0.04/0.3201
Fat mass (kg)	50.7 ± 7.9	48.12 (40.68–5.49)	0.09/0.0799	0.08/0.1153	0.09/0.0529	0.01/0.8573
Systolic blood pressure (mmHg)	133.9 ± 17.8	132.0 (120.0–143.0)	**0.18/<0.0001**	0.06/0.1944	**−0.12/0.0081**	**−0.22/<0.0001**
Diastolic blood pressure (mmHg)	76.6 ± 10.6	76.0 (70.0–81.0)	0.05/0.2091	−0.02/0.6255	−0.08/0.0692	**−0.10/0.0238**
Fasting glucose (mg/dL)	108.1 ± 36.3	98.0 (88.0–114.0)	−0.08/0.0796	**0.09/0.0388**	−0.07/0.0901	−0.01/0.9032
HbA1c (%)	6.2 ± 1.3	5.9 (5.4–6.5)	**0.17/<0.0001**	**0.16/0.0002**	−0.08/0.0716	0.05/0.2368
Total cholesterol (mg/dL)	184.6 ± 45.1	183.0 (155.0–210.0)	−0.06/0.1421	**0.10/0.0213**	**−0.11/0.0164**	**−0.15/0.0006**
LDL-cholesterol (mg/dL)	110.2 ± 35.7	110.0 (84.0–133.0)	−0.06/0.1533	**0.10/0.0241**	**−0.11/0.0157**	**−0.15/0.0009**
HDL-cholesterol (mg/dL)	42.8 ± 11.6	41.9 (35.0–49.0)	0.03/0.4388	**−0.14/0.0018**	−0.02/0.6179	−0.03/0.4563
Triglycerides (mg/dL)	160.2 ± 144.9	138.0 (100.0–188.0)	−0.02/0.5974	**0.13/0.0033**	0.03/0.4505	0.05/0.2355
Alanine aminotransferase (U/L)	51.3 ± 46.5	43.0 (33.0–58.0)	−0.04/0.3025	−0.05/0.2933	0.01/0.8602	0.04/0.3402
Aspartate aminotransferase (U/L)	30.1 ± 29.3	24.0 (19.0–32.0)	0.02/0.5842	0.01/0.7619	−0.03/0.4632	−0.04/0.3861
CRE (mg/dL)	0.86 ±0.2	0.83 (0.73–0.95)	0.02/0.7000	**0.37/<0.0001**	−0.02/0.6217	−0.02/0.6742
B_12_ (pg/mL)	334.5 ± 161.4	302.0 (245.0–380.0)	−0.08/0.0669	**−0.10/0.0177**	−0.03/0.4883	0.02/0.7315
Folic acid (ng/mL)	8.2 ± 3.6	7.5 (5.6–10.2)	**−0.20/<0.0001**	**−0.16/0.0002**	−0.04/0.2902	**0.10/0.0241**
Apnea Hypopnea Index	14.2 ± 21.2	5.5 (1.5–16.1)	**0.14/0.0024**	**0.16/0.0005**	−0.02/0.6504	**−0.09/0.0481**
IL-6 (pg/mL)	3.5 ± 4.7	2.5 (1.4–4.3)	**0.15/0.0016**	**0.14/0.0032**	−0.01/0.8205	**−0.11/0.0166**
ESR	17.7 ± 13.6	15.0 (8.0–24.0)	−0.02/0.6915	−0.07/0.1147	**0.17/<0.0001**	**0.16/0.0003**
CRP (g/L)	9.2 ± 12.5	6.4 (3.0–12.2)	**0.17/0.0001**	0.06/0.1488	0.02/0.6671	**−0.10/0.0247**
eGFR (CKD-EPI) mL/min/1.73 m^2^	92.0 ± 17.4	94.1 (80.4–104.6)	0.02/0.6020	**−0.33/<0.0001**	0.07/0.1049	0.04/0.3704
eGFR (MDRD) mL/min/1.73 m^2^	88.1 ± 18.9	87.6 (76.0–98.7)	0.02/0.6875	**−0.31/<0.0001**	0.04/0.3427	0.02/0.7079

* Spearman’s correlations, *p* values were considered significant when *p* > 0.05 (in bold). The values are given as the means ± standard deviations (SD) and median, interquartile range (IQR). Abbreviations: BMI, body mass index; CRE, creatinine; HbA1c, glycosylated hemoglobin; HDL-cholesterol, high-density lipoprotein cholesterol; LDL-cholesterol, low-density lipoprotein cholesterol; ESR, erythrocyte sedimentation rate; CRP, C-reactive protein; eGFR, estimated glomerular filtration rate. CKD-EPI, Chronic Kidney Disease Epidemiology Collaboration; MDRD, Modification of Diet in Renal Disease Study equation.

**Table 2:** Concentrations of asymmetric dimethylarginine (ADMA), symmetric dimethylarginine (SDMA), and arginine according to BMI, obesity level, hypertension, obstructive sleep apnea (OSA).

	N	ADMA, µmol/L		SDMA, µmol/L		Arginine, µmol/L		Ratio Arginine/ADMA, µmol/L	
		Mean ± SD	Median (IQR)	Mean ± SD	Median (IQR)	Mean ± SD	Median (IQR)	Mean ± SD	Median (IQR)
**BMI Level**									
I BMI 30.0–34.9	47	0.49 ± 0.09	0.49 (0.42–0.53)	1.24 ± 0.29	1.22 (1.06–1.38)	97.58 ± 21.54	91.27 (82.66–108.78)	176.75± 45.44	171.43 (141.44–200.94)
II BMI 35–39.9	148	0.51 ± 0.09	0.50 (0.45–0.56)	1.31 ± 0.37	1.27 (1.11–1.44)	103.68 ± 36.76	97.30 (86.68–112.80)	179.20± 65.85	168.34 (147.66–197.21)
III BMI ≥ 40	323	0.54 ± 0.09	0.53 (0.48-0.93)	1.35 ± 0.30	1.30 (1.15–1.51)	108.02 ± 41.60	102.18 (86.68–118.26)	176.40± 69.12	163.86 (138.02–197.16)
Statistics (Anova/Kruskal-Wallis tests)		F = 9.89; df = 2; *p* < **0.0001**	*p* < **0.0001**	F = 3.13; df = 2; *p* = **0.0443**	*p* = **0.0113**	F = 1.80; df = 2; *p* = 0.1664	*p* = 0.0716	F = 0.09; df = 2; *p* = 0.9126	*p* = 0.489
**Hypertension**									
No	169	0.52 ± 0.08	0.51 (0.47–0.56)	1.24 ± 0.29	1.27 (1.11–1.40)	110.20 ± 45.15	102.18 (90.70–117.68)	184.92± 75.84	171.30 (147.57–203.84)
Yes	349	0.53 ± 0.09	0.52 (0.46–0.59)	1.31 ± 0.37	1.34 (1.17–1.57)	103.78 ± 35.33	98.16 (83.81–114.24)	173.66 ± 60.86	164.23 (137.62–195.19)
Statistics (*t*-test/Mann-Whitney test)		*p* = 0.5904; *t* = 0.54	*p* = 0.6074	*p* = **0.0176**; *t* = 2.40	*p* = **0.0500**	*p* = 0.0781; *t* =−1.76	*p* = **0.0152**	*p* = 0.0697; *t* =−1.82	*p* = **0.0325**
**AHI Level**									
AHI < 5	276	0.51 ± 0.09	0.50 (0.45–0.57)	1.29 ± 0.33	1.27 (1.11–1.40)	106.31 ± 37.22	99.31 (86.68–116.53)	181.83 ± 62.73	173.85 (140.16–206.75)
AHI ≥ 5	242	0.54 ± 0.09	0.53 (0.48–0.59)	1.37 ± 0.32	1.32 (1.17–1.57)	103.53 ± 41.05	100.17 (86.68–113.09)	173.74 ± 68.39	159.56 (138.71–191.45)
Statistics (*t*-test/Mann-Whitney test)		*p* = **0.0036**; *t* = −2.93	*p* = **0.00072**	*p* = **0.0041**; *t* = −2.89	*p* = **0.00117**	*p* = 0.9508; *t* = −0.06	*p* = 0.6749	*p* = 0.1878; *t* = 1.32	*p* = **0.0168**

*p*-value < 0.05 are in bold.

**Table 3:** Concentrations of ADMA, SDMA and arginine according to *NOS3* G894T and *NOS3* 4a/4b genotypes.

N		ADMA, µmol/L		SDMA, µmol/L		Arginine, µmol/L	
		Mean ± SD	Median (IQR)	Mean ± SD	Median (IQR)	Mean ± SD	Median (IQR)
**Genotype *NOS3* G894T**							
GT	150	0.50 ± 0.09	0.49 (0.45-0.55)	1.28 ± 0.27	1.24 (1.09–1.44)	104.38 ± 30.25	102.18 (86.68–115.96)
TT	83	0.53 ± 0.09	0.51 (0.46–0.59)	1.40 ± 0.47	1.32 (1.13–1.53)	106.13 ± 46.30	95.87 (83.24–114.24)
GG	285	0.53± 0.09	0.53 (0.47–0.59)	1.34 ± 0.29	1.29 (1.15–1.46)	106.59 ± 40.70	99.60 (86.68–116.53)
Statistics (Anova/Kruskal-Wallis tests)		F = 6.13; df = 2; *p* = **0.0023**	*p* = **0.0019**	F = 3.77; df = 2; *p* = **0.0237**	*p* = 0.0630	F = 0.16; df = 2; *p* = 0.8515	*p* = 0.3947
**Genotype *NOS3* 4a/4b**							
4a/4a	19	0.50 ± 0.08	0.51 (0.44–0.55)	1.30 ± 0.28	1.33 (1.09–1.47)	106.47 ± 25.97	99.31 (88.98–116.53)
4a/4b	158	0.54 ± 0.10	0.53 (0.47–0.59)	1.35 ± 0.29	1.31 (1.16–1.49)	109.66 ± 44.49	102.18 (86.11–117.11)
4b/4b	341	0.52 ± 0.08	0.51 (0.46–0.58)	1.32 ± 0.33	1.28 (1.11–1.45)	104.17 ± 36.61	98.74 (86.11–114.24)
Statistics (Anova/Kruskal-Wallis tests)		F = 2.29; df = 2; *p* = 0.1020	*p* = 0.3073	F = 0.41; df = 2; *p* = 0.6670	*p* = 0.4418	F = 1.08; df = 2; *p* = 0.3410	*p* = 0.401

*p*-value < 0.05 are in bold.

**Table 4:** Logistic regression analyses for factors associated with obstructive sleep apnea (OSA).

Variables	OSA		Model 1 ^a^ OR (95% CI)	*p* ^a^	Model 2 ^b^ OR (95% CI)	*p* ^b^
	AHI < 5 *n* = 276	AHI ≥ 5 *n* = 242				
ADMA above median (>0.514 µmol/L), *n* (%) Yes No	117 (42.5) 159 (57.6)	135 (55.8) 107 (44.2)	1.71 (1.21–2.43)	**0.002**	**1.74 (1.12–2.71)**	**0.0132**
SDMA above median (>1.286 µmol/L), *n* (%) Yes No	119 (43.0) 157 (57.0)	132 (54.5) 110 (45.5)	1.58 (1.12–2.24)	**0.010**	**1.15 (0.73–1.81)**	0.537
Age above median (>43 years), *n* (%) Yes No	97 (35.2) 179 (64.8)	145 (59.9) 97 (40.1)	2.76 (1.93–3.94)	**<0.001**	**2.25 (1.42–3.56)**	**<0.001**
Hypertension, *n* (%) Yes No	147 (53.3) 129 (46.7)	187 (77.3) 55 (22.7)	2.98 (2.03–4.37)	**<0.001**	**1.77 (1.11–2.80)**	**0.015**
BMI, *n* (%) above 40 30–39.9	149 (54.0) 127 (46.0)	172 (71.0) 70 (29.0)	2.09 (1.45–3.02)	**<0.001**	**2.26 (1.50–3.50)**	**<0.001**
Sex, *n* (%) Male Female	36 (13.0) 240 (87.0)	98 (40.5) 144 (59.5)	4.54 (2.94–7.00)	**<0.001**	**3.82 (2.30–6.34)**	**<0.001**

^a^ Model 1, ORs without adjustment; ^b^ Model 2, ORs adjusted for Model 1 plus smoking status, dyslipidemia, diabetes, eGFR > 60 mL/min/1.73 m^2^. *p*-value < 0.05 are in bold. OSA; obstructive sleep apnea; OR, odds ratio; CI, confidence interval.

## References

[1] Osman A.M., Carter S.G., Carberry J.C., Eckert D.J. (2018). Obstructive sleep apnea: current perspectives. Nat. Sci. Sleep.

[2] Liu X., Xu X., Shang R., Chen Y. (2018). Asymmetric dimethylarginine (ADMA) as an important risk factor for the increased cardiovascular diseases and heart failure in chronic kidney disease. Nitric Oxide Biol. Chem..

[3] Tsikas D., Boger R.H., Sandmann J., Bode-Boger S.M., Frolich J.C. (2000). Endogenous nitric oxide synthase inhibitors are responsible for the L-arginine paradox. FEBS Lett..

[4] Sasser J.M., Moningka N.C., Cunningham M.W., Croker B., Baylis C. (2010). Asymmetric dimethylarginine in angiotensin II-induced hypertension. Am. J. Physiol. Regul. Integr. Comp. Physiol..

[5] Maas R., Boger R., Luneburg N. (2009). ADMA and the role of the genes: lessons from genetically modified animals and human gene polymorphisms. Pharmacol. Res..

[6] Tain Y.L., Hsu C.N. (2017). Toxic Dimethylarginines: Asymmetric Dimethylarginine (ADMA) and Symmetric Dimethylarginine (SDMA). Toxins.

[7] Leiper J., Vallance P. (1999). Biological significance of endogenous methylarginines that inhibit nitric oxide synthases. Cardiovasc. Res..

[8] Xuan C., Liu Z.F., Wang Q., Guo F.F., Zhang X., He G.W., Lun L.M. (2017). Increased serum concentrations of asymmetric dimethylarginine (ADMA) in patients with early-onset coronary artery disease. Clin. Chim. Acta.

[9] Wrzosek M., Sokal M., Sawicka A., Wlodarczyk M., Glowala M., Wrzosek M., Kosior M., Talalaj M., Biecek P., Nowicka G. (2015). Impact of obesity and nitric oxide synthase gene G894T polymorphism on essential hypertension. J. Physiol. Pharmacol..

[10] (2010). Standards of medical care in diabetes—2010. Diabetes Care.

[11] (2002). Third Report of the National Cholesterol Education Program (NCEP) Expert Panel on Detection, Evaluation, and Treatment of High Blood Cholesterol in Adults (Adult Treatment Panel III) final report. Circulation.

[12] Mbata G., Chukwuka J. (2012). Obstructive sleep apnea hypopnea syndrome. Ann. Med. Health Sci. Res..

[13] Al-Maqbali S.R., Mula-Abed W.A. (2014). Comparison between Three Different Equations for the Estimation of Glomerular Filtration Rate in Omani Patients with Type 2 Diabetes Mellitus. Sultan Qaboos Univ. Med J..

[14] Desjardins F., Balligand J.L. (2006). Nitric oxide-dependent endothelial function and cardiovascular disease. Acta Clin. Belg..

[15] Dobutovic B., Smiljanic K., Soskic S., Düngen H.-D., Isenovi E.R. (2011). Nitric Oxide and Its Role in Cardiovascular Diseases. Open Nitric Oxide J..

[16] Koren D., Chirinos J.A., Katz L.E., Mohler E.R., Gallagher P.R., Mitchell G.F., Marcus C.L. (2015). Interrelationships between obesity, obstructive sleep apnea syndrome and cardiovascular risk in obese adolescents. Int. J. Obes..

[17] Spoto B., Parlongo R.M., Parlongo G., Sgro E., Zoccali C. (2007). The enzymatic machinery for ADMA synthesis and degradation is fully expressed in human adipocytes. J. Nephrol..

[18] Sydow K., Fortmann S.P., Fair J.M., Varady A., Hlatky M.A., Go A.S., Iribarren C., Tsao P.S. (2010). Distribution of asymmetric dimethylarginine among 980 healthy, older adults of different ethnicities. Clin. Chem..

[19] Hao J., Zhang Y., Yan X., Yan F., Sun Y., Zeng J., Waigel S., Yin Y., Fraig M.M., Egilmez N.K. (2018). Circulating Adipose Fatty Acid Binding Protein Is a New Link Underlying Obesity-Associated Breast/Mammary Tumor Development. Cell Metab..

[20] Mangiacapra F., Conte M., Demartini C., Muller O., Delrue L., Dierickx K., Di Sciascio G., Trimarco B., De Bruyne B., Wijns W. (2016). Relationship of asymmetric dimethylarginine (ADMA) with extent and functional severity of coronary atherosclerosis. Int. J. Cardiol..

[21] Xuan C., Tian Q.W., Li H., Zhang B.B., He G.W., Lun L.M. (2016). Levels of asymmetric dimethylarginine (ADMA), an endogenous nitric oxide synthase inhibitor, and risk of coronary artery disease: A meta-analysis based on 4713 participants. Eur. J. Prev. Cardiol..

[22] Triches C.B., Mayer S., Quinto B.M.R., Batista M.C., Zanella M.T. (2018). Association of endothelial dysfunction with cardiovascular risk factors and new-onset diabetes mellitus in patients with hypertension. J. Clin. Hypertens..

[23] Sciacqua A., Grillo N., Quero M., Sesti G., Perticone F. (2012). Asymmetric dimethylarginine plasma levels and endothelial function in newly diagnosed type 2 diabetic patients. Int. J. Mol. Sci..

[24] Hammes M.S., Watson S., Coe F.L., Ahmed F., Beltran E., Dhar P. (2015). Asymmetric dimethylarginine and whole blood viscosity in renal failure. Clin. Hemorheol. Microcirc..

[25] Boelaert J., Schepers E., Glorieux G., Eloot S., Vanholder R., Lynen F. (2016). Determination of Asymmetric and Symmetric Dimethylarginine in Serum from Patients with Chronic Kidney Disease: UPLC-MS/MS versus ELISA. Toxins.

[26] Glantz H., Thunstrom E., Herlitz J., Cederin B., Nasic S., Ejdeback J., Peker Y. (2013). Occurrence and predictors of obstructive sleep apnea in a revascularized coronary artery disease cohort. Ann. Am. Thorac. Soc..

[27] Drager L.F., Genta P.R., Pedrosa R.P., Nerbass F.B., Gonzaga C.C., Krieger E.M., Lorenzi-Filho G. (2010). Characteristics and predictors of obstructive sleep apnea in patients with systemic hypertension. Am. J. Cardiol..

[28] Badran M., Abuyassin B., Golbidi S., Ayas N., Laher I. (2016). Uncoupling of Vascular Nitric Oxide Synthase Caused by Intermittent Hypoxia. Oxidative Med. Cell. Longev..

[29] Tsioufis C., Dimitriadis K., Andrikou E., Thomopoulos C., Tsiachris D., Stefanadi E., Mihas C., Miliou A., Papademetriou V., Stefanadis C. (2010). ADMA, C-reactive protein, and albuminuria in untreated essential hypertension: A cross-sectional study. Am. J. Kidney Dis..

[30] Zhang Z., Yang Y., Hill M.A., Wu J. (2012). Does C-reactive protein contribute to atherothrombosis via oxidant-mediated release of pro-thrombotic factors and activation of platelets?. Front. Physiol..

[31] Thomopoulos C., Tsioufis C., Dimitriadis K., Tsiachris D., Tousoulis D., Manolis A., Alchanatis M., Kallikazaros I., Stefanadis C. (2008). Obstructive sleep apnoea syndrome is associated with enhanced sub-clinical inflammation and asymmetric dimethyl-arginine levels in hypertensives. J. Hum. Hypertens..

[32] Korandji C., Zeller M., Guilland J.C., Vergely C., Sicard P., Duvillard L., Gambert P., Moreau D., Cottin Y., Rochette L. (2007). Asymmetric dimethylarginine (ADMA) and hyperhomocysteinemia in patients with acute myocardial infarction. Clin. Biochem..

[33] Sydow K., Schwedhelm E., Arakawa N., Bode-Böger S.M., Tsikas D., Hornig B., Frölich J., Böger R.H. (2003). ADMA and oxidative stress are responsible for endothelial dysfunction in hyperhomocyst(e)inemia: effects of L-arginine and B vitamins. Cardiovasc. Res..

[34] Yoo J.H., Lee S.C. (2001). Elevated levels of plasma homocyst(e)ine and asymmetric dimethylarginine in elderly patients with stroke. Atherosclerosis.

[35] Doshi S., McDowell I., Goodfellow J., Stabler S., Boger R., Allen R., Newcombe R., Lewis M., Moat S. (2005). Relationship between S-adenosylmethionine, S-adenosylhomocysteine, asymmetric dimethylarginine, and endothelial function in healthy human subjects during experimental hyper- and hypohomocysteinemia. Metabolism.

[36] Boger R.H., Lentz S.R., Bode-Boger S.M., Knapp H.R., Haynes W.G. (2001). Elevation of asymmetrical dimethylarginine may mediate endothelial dysfunction during experimental hyperhomocyst(e)inaemia in humans. Clin. Sci..

[37] Perticone F., Sciacqua A., Maio R., Perticone M., Maas R., Boger R.H., Tripepi G., Sesti G., Zoccali C. (2005). Asymmetric dimethylarginine, L-arginine, and endothelial dysfunction in essential hypertension. J. Am. Coll. Cardiol..

[38] Sonmez A., Celebi G., Erdem G., Tapan S., Genc H., Tasci I., Ercin C.N., Dogru T., Kilic S., Uckaya G. (2010). Plasma apelin and ADMA Levels in patients with essential hypertension. Clin. Exp. Hypertens..

